# Supplementary material for: Skilful prediction of Sahel summer rainfall on inter-annual and multi-year timescales
Source: Nat Commun. 2017 May 25;8:14966. doi: 10.1038/ncomms14966 (PMC5529672; doi:10.1038/ncomms14966)
Supplement: Supplementary Information — Supplementary Figures, Supplementary Notes and Supplementary References [file ncomms14966-s1.pdf]

Supplementary Figures

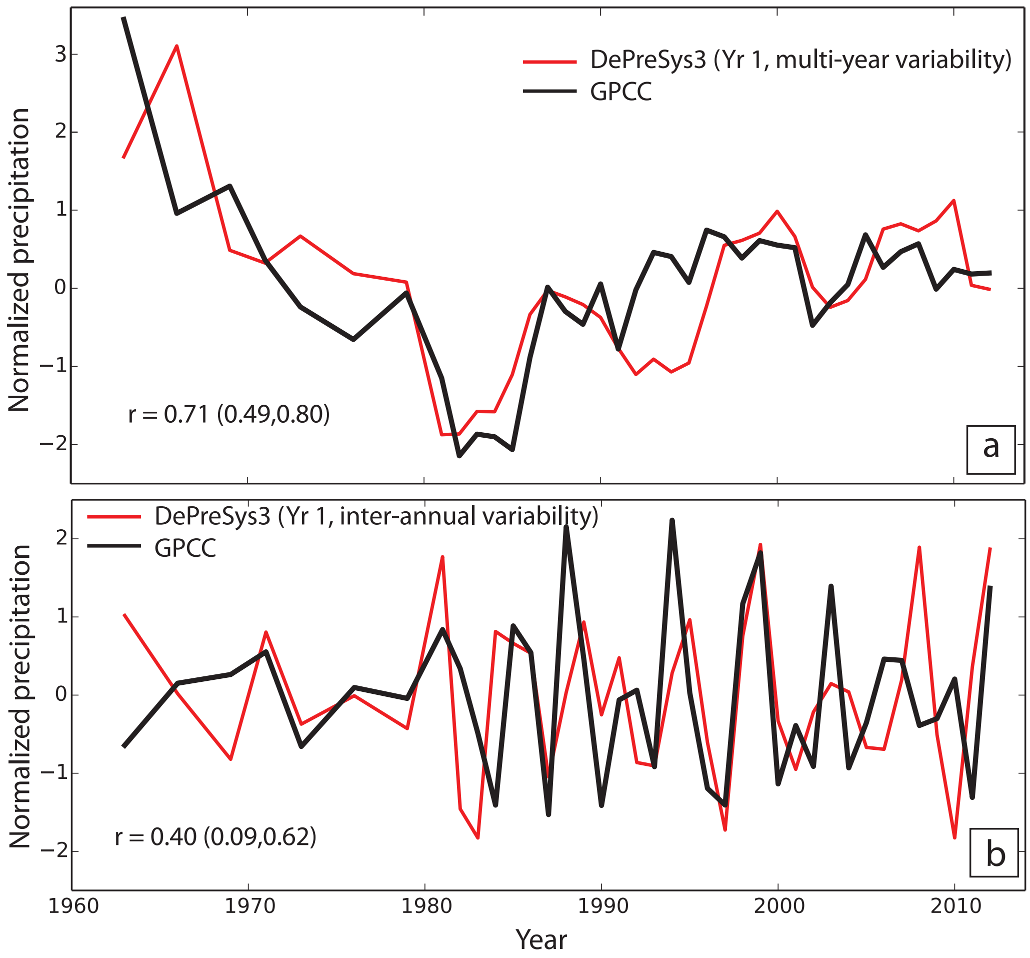

Supplementary Figure 1 **Multi-year and inter-annual components of year one lead hindcasts, as used in the moisture budget analysis.** (a) Multi-year component (i.e smoothed with a 5 year running mean) of DePreSys3 Yr1 hindcasts and observations (see Methods). Timeseries are linearly de-trended and normalised. (b) As for panel (a) but for inter-annual component (i.e. residual). Correlations and 5–95 % confidence intervals computed from re-sampling and block bootstrapping are indicated.

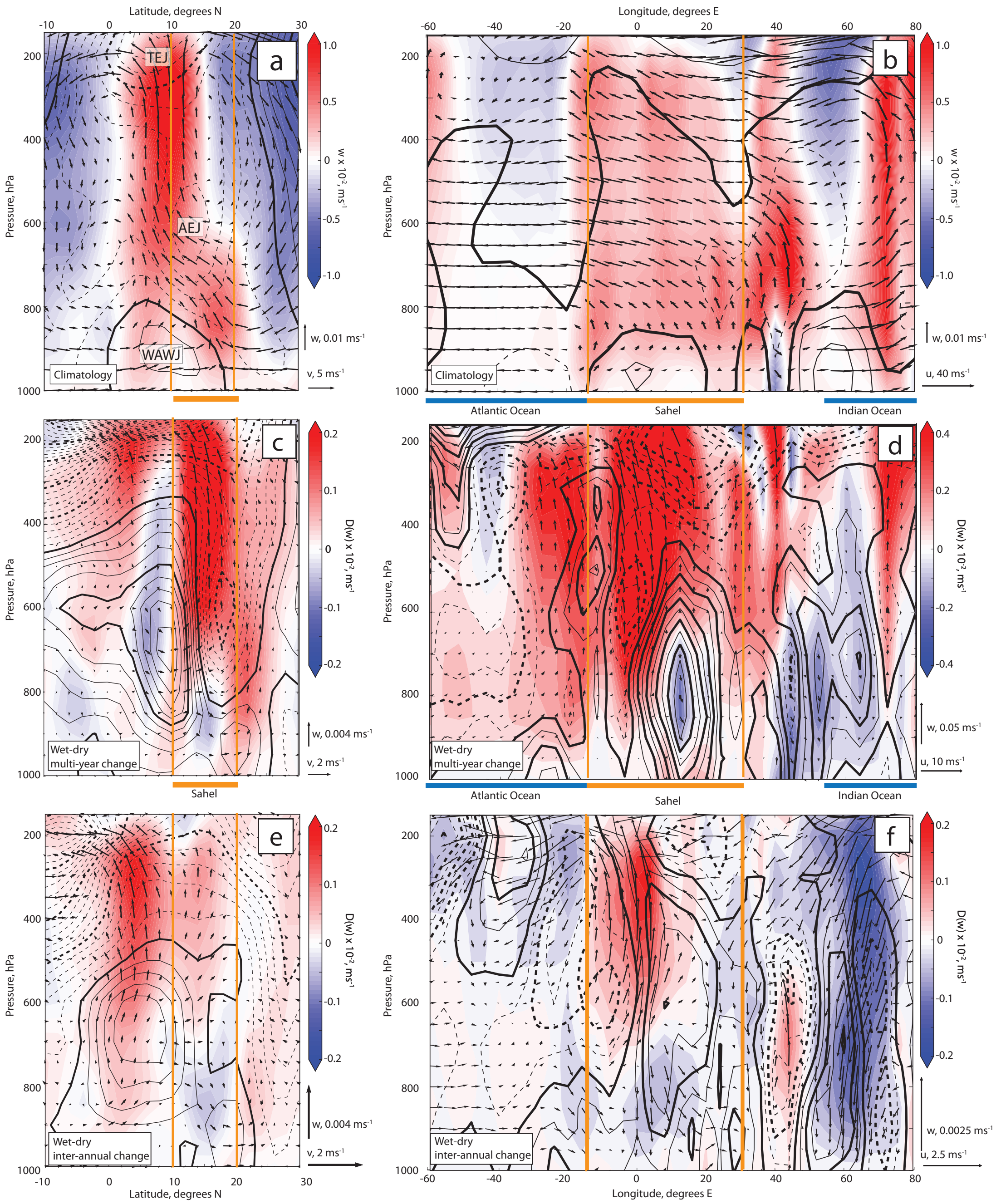

Supplementary Figure 2 **Circulation patterns associated with Sahel rainfall in re-analysis data** As for Fig. 3 in the main text but using NCEP/NCAR and JRA-55 re-analysis data (averaged). DePreSys3 precipitation time series have been used to produce wet and dry composites.

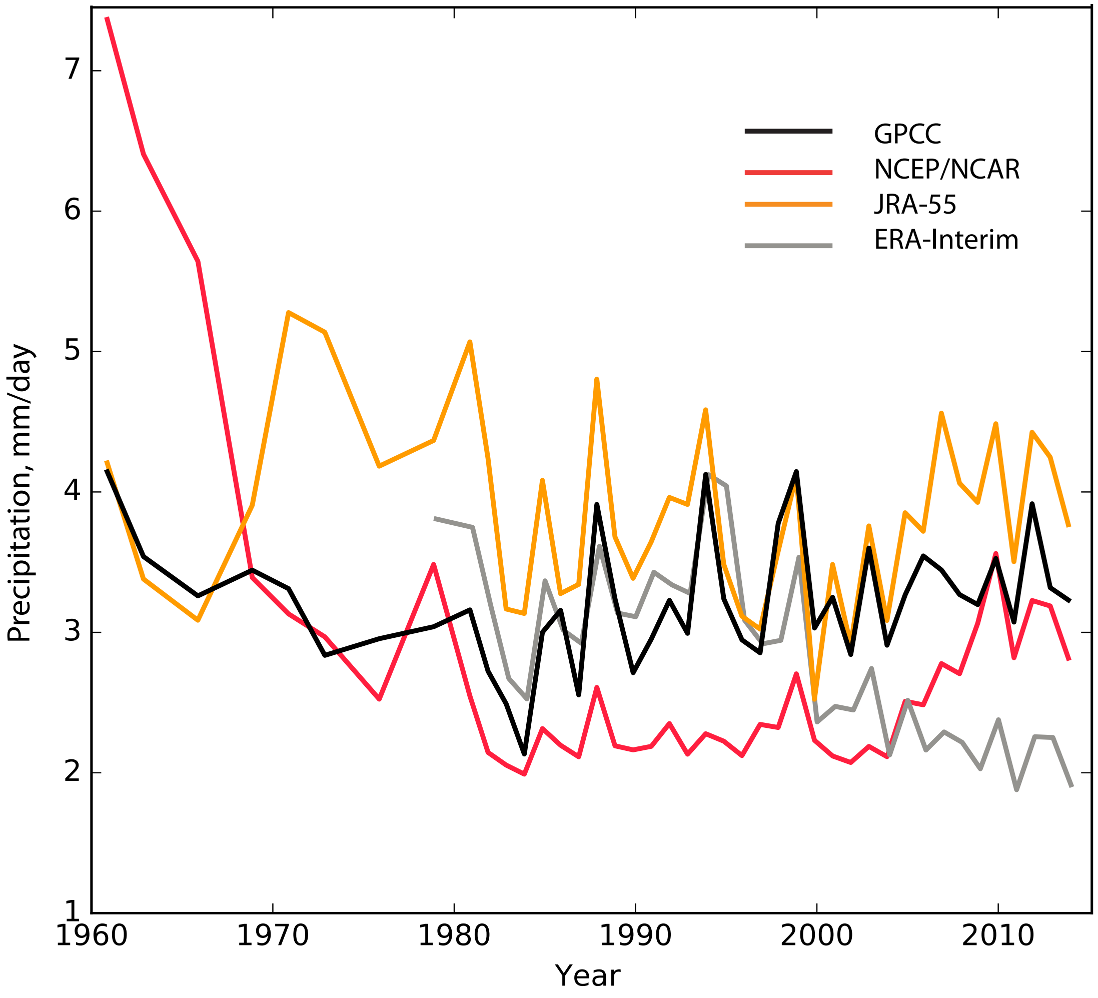

Supplementary Figure 3 **Limitations in re-analysis Sahel summer precipitation** Timeseries of Sahel summer precipitation from GPCC observations and three different reanalysis products. Data has not been detrended. Note, the ERA-Interim data is from the European Centre for Medium-Range Weather Forecasts (ECMFW) Interim Re-Analysis (available post-1979) [10]

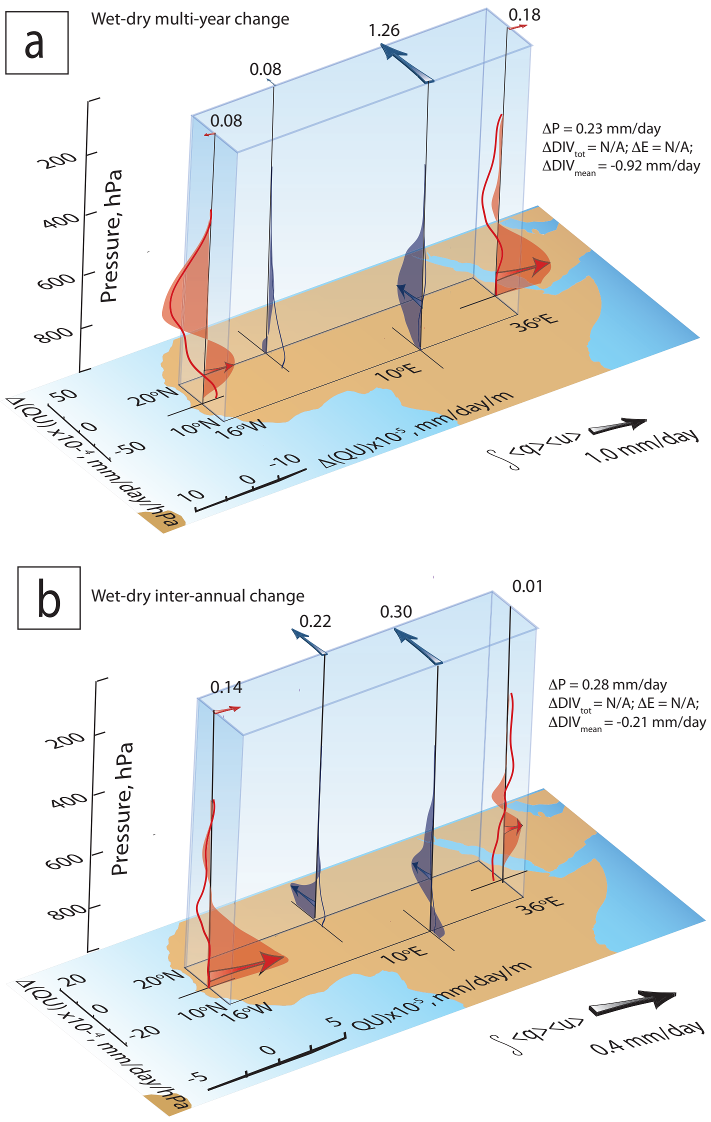

Supplementary Figure 4 **Changes in moisture fluxes between wet and dry Sahel summers from re-analysis data.** (a) As for Fig. 4a in main text but using specific humidity, wind, and surface pressure data from NCAR/NCEP re-analysis (see Methods). DePreSys3 rainfall levels are utilised to determine weights for averaged wet and dry composites. All divergence, recycling rations and flux estimates are for the mean flow only i.e. $\langle q\mathbf{u}\rangle$ was not available in the re-analysis dataset. (b) As for panel (a) but for inter-annual variability.

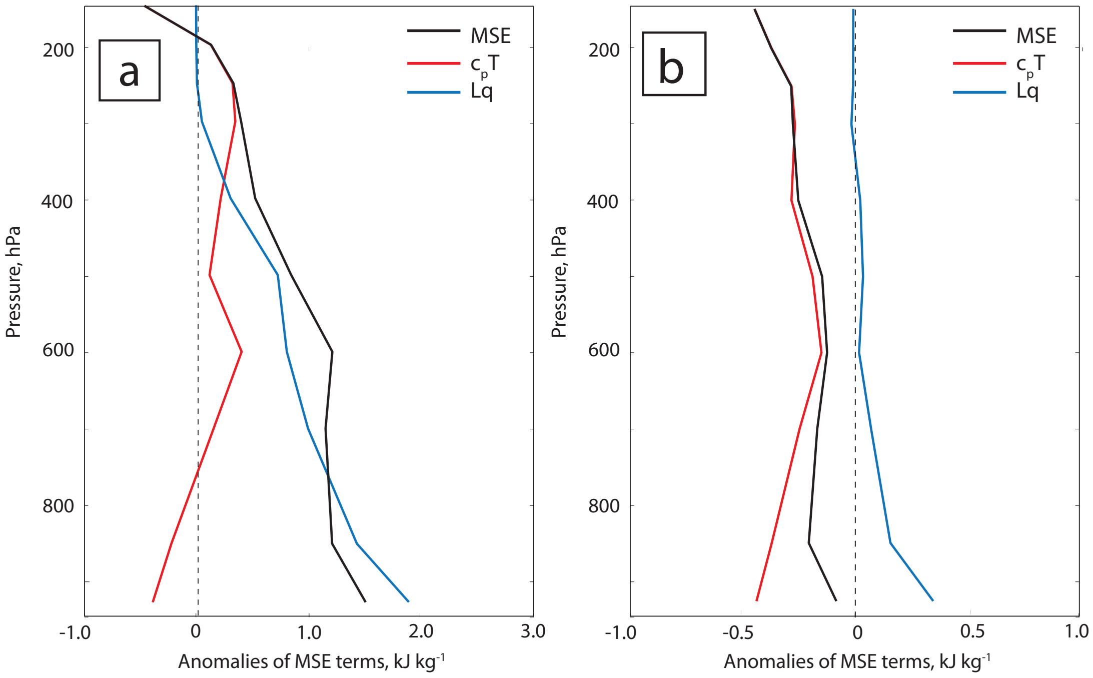

Supplementary Figure 5 **Drivers of atmospheric stability in re-analysis data** As for Fig. 5 but for but for average of NCEP/NCAR and JRA-55 re-analysis. Wet and dry composites determined using the DePreSys3 precipitation data.

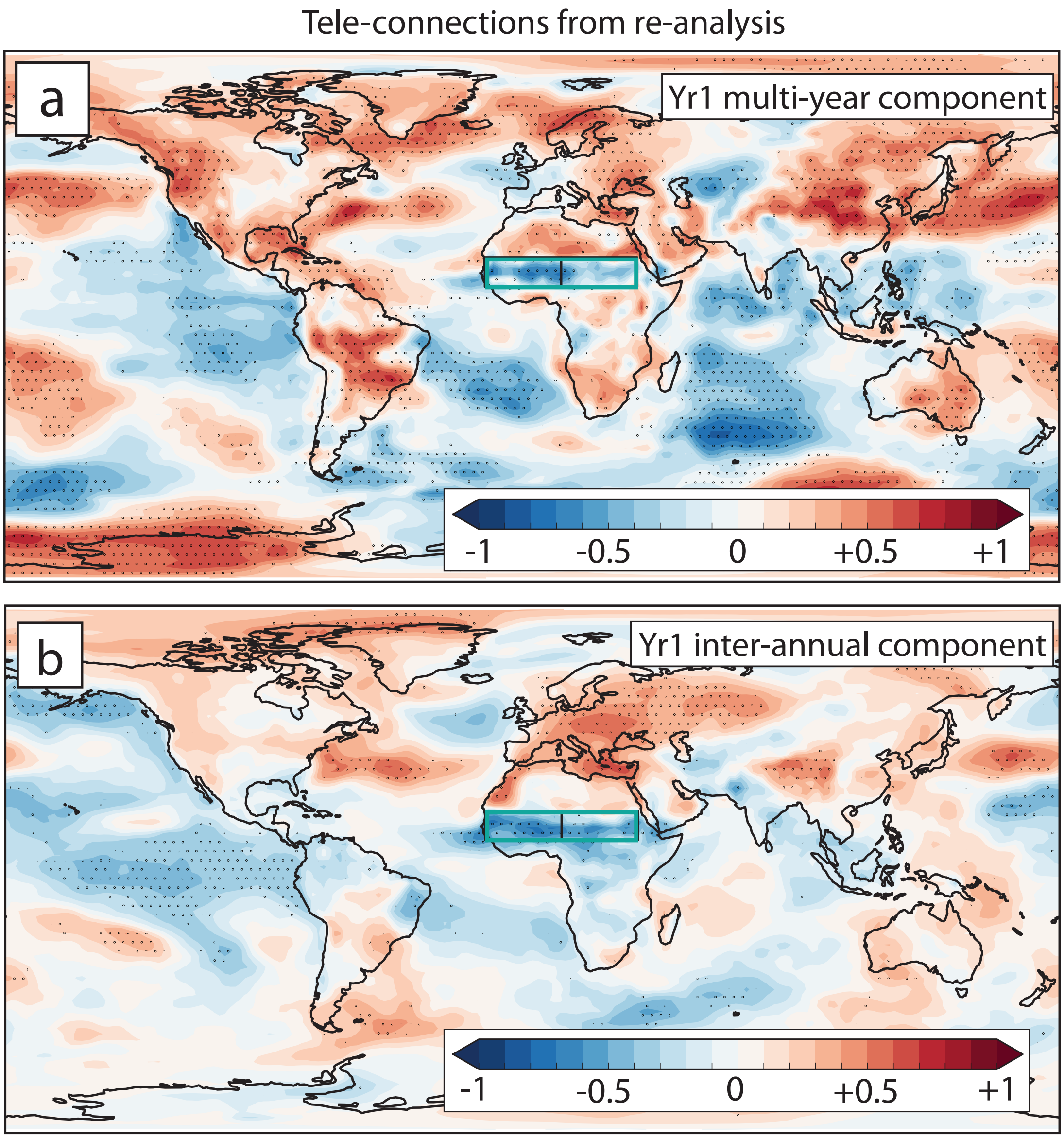

Supplementary Figure 6 **Relationship between global surface temperature patterns and Sahel rainfall in re-analysis** (a) Correlation between Sahel summer rainfall (GPCC data) and re-analysis global surface temperatures for multi-year variability (both detrended). Data is average correlation for NCAR/NCEP and JRA-55 products. Stippled areas show where both data products show significance correlation. (b) As for (a) but for inter-annual variability. Compare with Figs. 6a & 6b.

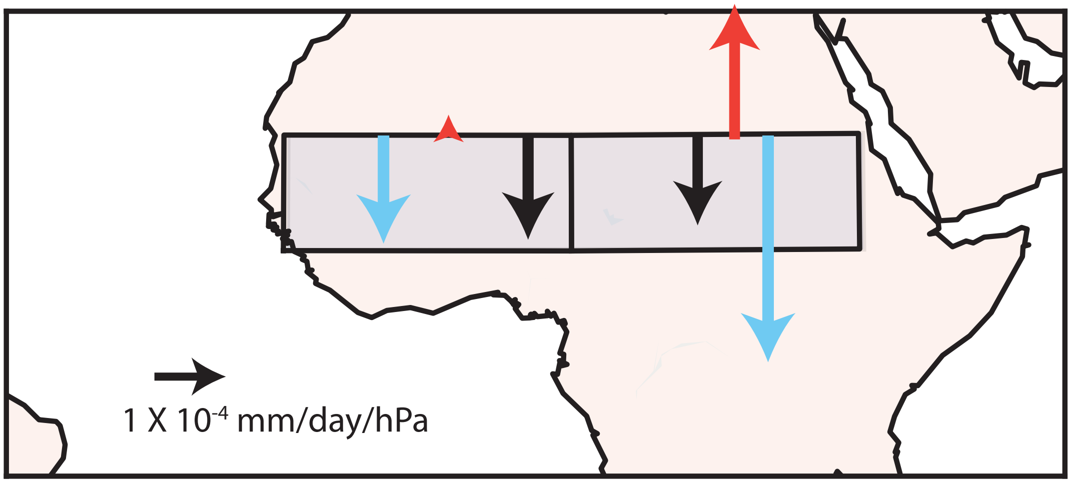

Supplementary Figure 7 **Changes in low level moisture flux across the northern Sahel boundary on multi-year timescales** Black arrows show moisture flux change, $\Delta <\mathrm{q}><\mathrm{u}>$, between wet and dry years at 925 hPa along the northern Sahel boundary. Moisture flux changes have been partitioned into the west and east Sahel (delineated by 10° E). Blue arrows indicate contribution from specific humidity changes, $<\mathrm{u}>\Delta<\mathrm{q}>$, and red arrows show contribution to the external moisture flux change from wind changes, $<\mathrm{q}>\Delta<\mathrm{u}>$. Black boxes show west and east Sahel regions. Note how increased humidity in low level winds sourced from the Mediterranean counteract the weakening of these northerly winds, particularly in the eastern Sahel. Data is for multi-year component of one year lead time hindcasts.

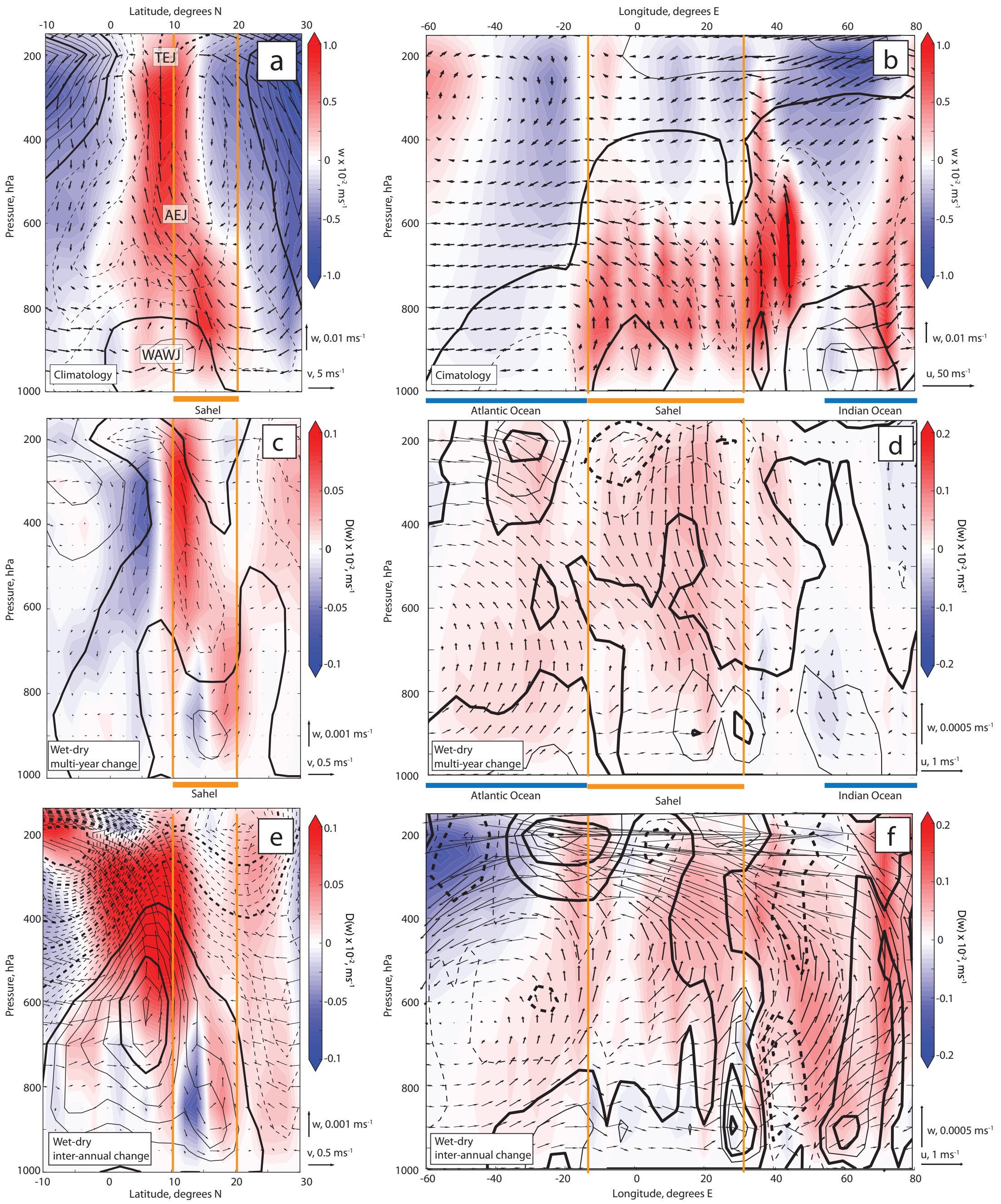

Supplementary Figure 8 **Impacts of north Atlantic SST and ENSO on Sahel summer circulation patterns**

As for Fig. 3 in main text but composites in panels c & d are computed using a warm-cold north Atlantic SST (25 to 60° N, 7 to 70° E), and composites in panels e & f are based on an ENSO index: a cold-warm south east Pacific SST (-5 to 5° N, -170 to -120° E)

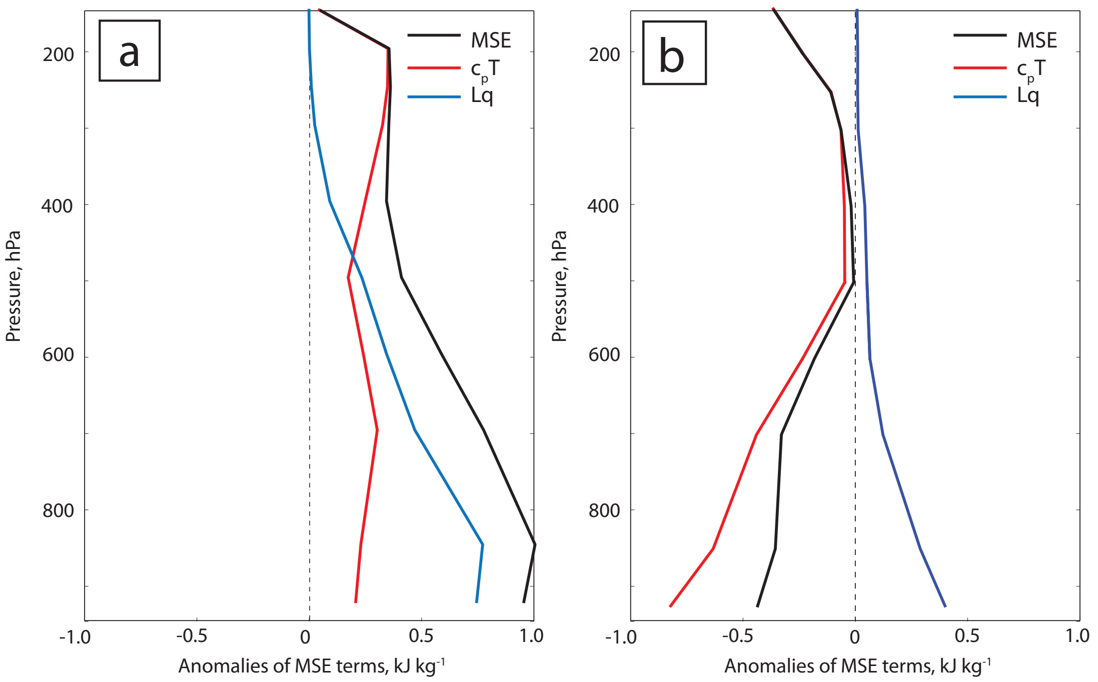

Supplementary Figure 9 **Impacts of north Atlantic SST and ENSO on summer atmospheric stability over the Sahel** (a) As for Fig. 5a in main text but for but composites are based on a warm-cold north Atlantic SST (25 to 60° N, 7 to 70° E). (b) As for Fig. 5b but for but composites are based on an ENSO index: a cold-warm south east Pacific SST (-5 to 5° N, -170 to -120° E).

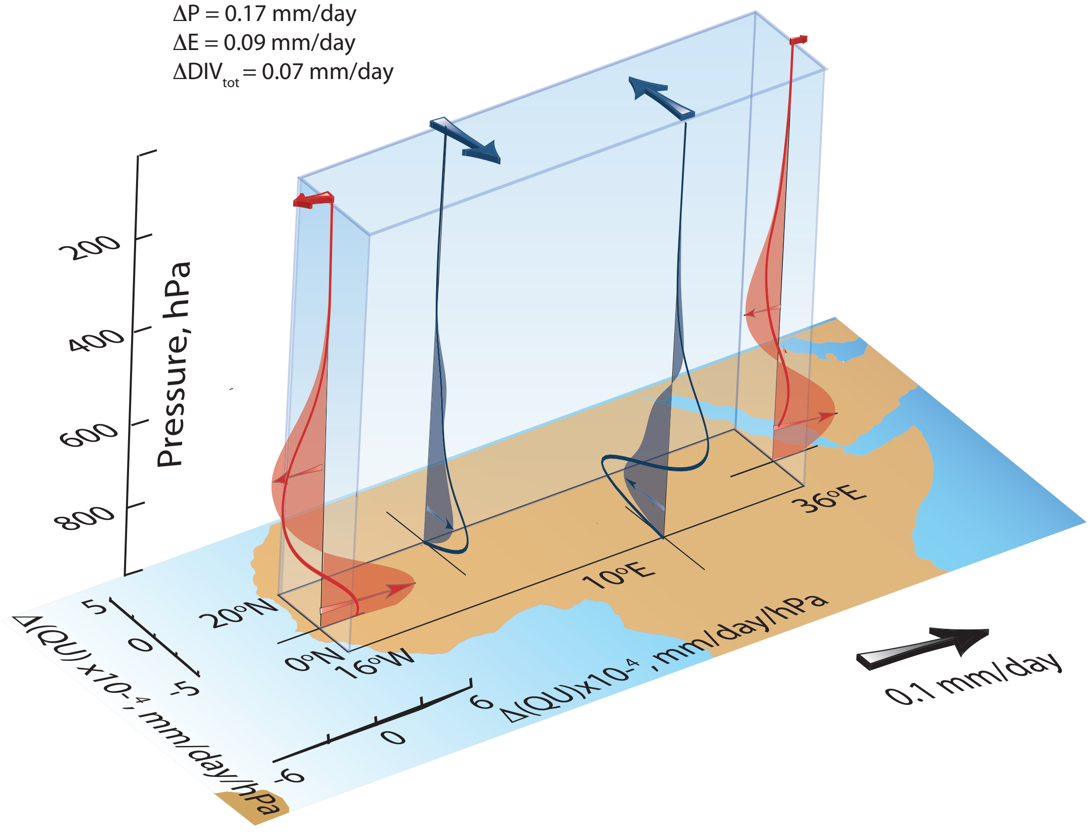

Supplementary Figure 10 **Changes in moisture fluxes between wet and dry Sahel summers for hindcasts averaged 2–5 years from initialization.** As for Fig. 4a in main text but for DePreSys3 hindcasts averaged over years 2-5 from initialization (shows multi-year change).

Supplementary Tables

Supplementary Table 1 Correlations between de-trended observed Sahel precipitation and various CMIP5 initialized model outputs[1]. Values are computed for the Sahel box and the West and East Sahel (delineated by 10°E). Start years are as in DePreSys3, but only up to 2009 (the end of the CMIP5 hindcasts), such that values are slightly different to Fig. 1. Models analysed are all initialized: CanCM4, GFDL, MPI and MIROC5 have January start dates, other models have November start dates (i.e. 2 months earlier). Results are from a 10 member model mean, other than MPI and MIROC5, with 3 and 6 members, respectively. For the multi-model mean, differences in ensemble size are accounted for by bootstrapping - the 49 CMIP5 members are randomly re-sampled to 5000 ten-member sub-sets and compared with different combinations of the DePreSys3 ten members (resampled with replacement). c denotes a significantly higher correlation than climatology (i.e. $r = 0$) at the 90% level. d means that DePreSys3 has significantly higher correlation at the 90% level. Persistence skill is also included, computed by persisting the observed average over the equivalent number of summer seasons for the period prior to each model initialisation date.

	**Low frequency variability (lead-times: 2–5 yrs)**			**Inter-annual variability (lead-times: 1 yr)**		
	**Whole Sahel**	**West Sahel**	**East Sahel**	**Whole Sahel**	**West Sahel**	**East Sahel**
**DePreSys3**	0.70c	0.71c	0.62c	0.45c	0.48c	0.29c
**CMIP5**	0.54c	0.54c	0.42c	0.28c	0.29c	0.20
**DePreSys1A** [2]	0.44	0.55c	0.18d	0.11d	0.11d	0.04d
**DePreSys1F** [2]	0.64c	0.67c	0.51c	0.17d	0.13d	0.23
**CanCM4** [3]	0.69c	0.64c	0.59c	0.32c	0.39c	0.21
**GFDL** [4]	0.54c	0.61c	0.34	0.33c	0.38c	0.22
**MPI** [5]	0.15	0.19d	0.08	0.25	0.26	0.22
**MIROC5** [6]	0.70c	0.63c	0.66c	0.21	0.17	0.19
**Persistence**	0.50	0.49	0.40	0.21	0.34	0.03

Supplementary Note 1: Comparison of DePreSys3 circulation patterns and moisture fluxes with re-analysis data

In the main text, we show the summer climatological circulation patterns over the Sahel, along with the circulation changes associated with Sahel rainfall change on both multi-year and inter-annual timescales (Fig. 3). Here, we perform a similar analysis using the average of NCEP/NCAR [7] and JRA-55 [8,9] re-analyses (Supplementary Figure 2).

We find that the atmospheric climatology is largely consistent between DePreSys3 and the re-analysis products (i.e. compare Figs. 3a & 3b with Supplementary Figures 2a & 2b). One subtle difference is the position of the African Easterly Jet, which sits slightly further to the north in the re-analysis. In addition, the deep circulation cell tends to extend further to the north (and south) in the re-analysis, supporting our discussions around Figure 10. Due to this climatological northward extension, the re-analysis data show enhanced ascent at upper levels in the zonal circulation section when compared to DePreSys3 (Fig. 3b & Supplementary Figure 2b).

Examination of the re-analysis wet minus dry composites show that Sahel rainfall is associated with similar circulation shifts as in DePreSys3: on multi-year timescales the meridional circulation pattern is clearly migrated northward during wet years and the AEJ and WAWJ strengthen; on inter-annual timescales, the deep convection cell is strengthened and the TEJ enhanced (Supplementary Figure 2). However, we do note that enhanced upwelling at lower levels on inter-annual timescales is not apparent in the re-analysis data (i.e. compare Fig. 3f & Supplementary Figure 2f). This discrepancy is likely a reflection of the fact that the climatologically the main rainbelt in DePreSys3 is shifted to the south (Fig. 10). Furthermore, the re-analysis data indicates that in addition to a strengthening, there is also a hint of a meridional migration of the Sahelian deep circulation cell on inter-annual timescales.

We note that DePreSys3 precipitation time series have been used in this section to produce wet and dry composites to assess the observed mechanisms consistent with the forecast rainfall. We chose not to use precipitation estimates from the re-analysis data because they show large inconsistencies with observations (see Methods & Supplementary Figure 3). Repeating this analysis with composites based on the GPCC precipitation timeseries produced

similar results, reflecting the good rainfall skill of DePreSys3

In the main text, we compute column integrals of moisture flux using $\int\langle q\mathbf{u}\rangle$ which is computed exactly at each model timestep and level in DePreSys3 (see Methods). However, only $\langle q\rangle$ and $\langle \mathbf{u}\rangle$ are available for re-analysis data. We therefore use the discrete interval, $\int\langle q\rangle\langle \mathbf{u}\rangle = 1/(\rho_{\mathrm{w}}\mathrm{g})\sum_{0}^{\ln \mathrm{p_s}} \mathrm{p}\langle \mathrm{q}\rangle\langle \mathbf{u}\rangle\Delta(\ln \mathrm{p})$, where $\langle\rangle$ represents the summer mean, g is the acceleration due to gravity, ρ_w is the density of water and p_s is taken to be the surface pressure level [11]. As values of $\langle q\rangle$ and $\langle \mathbf{u}\rangle$ are only available at discreet pressure levels, values are interpolated throughout the atmosphere using a cubic spline onto 10 hPa spaced pressure levels before integration. Unlike integrals of $\int\langle q\mathbf{u}\rangle$, columnar moisture flux calculated using $\langle q\rangle\langle \mathbf{u}\rangle$ represents the contribution of moisture flux changes due to the monthly mean circulation and do not encompass variability due to the intra-monthly transient eddy flow [12].

Hence, estimates of moisture fluxes from re-analysis data are imperfect. Nevertheless, moisture flux changes between wet and dry periods around the Sahel domain for re-analysis data reveal broadly the same signatures as DePreSys3 (Figs. 5 & Supplementary Figure 4). On multi-year timescales, moisture flux divergence due to the mean flow is dominated by monsoon flow from the south and is much larger than divergence anomalies on yearly timescales. Increased westerly winds at 850 hPa, carrying moisture from the Atlantic are evident on both timescales across the Sahel. Like the DePreSys3 output, the southward flowing surface Harmattan winds carry more moisture during wetter summers, although in the re-analysis data weakened wind strengths overcome this signal in the total moisture flux (particularly on inter-annual timescales). In general, moisture flux anomalies are larger in the re-analysis data compared to DePreSys3, particularly along the southern boundary of the Sahel on multi-year timescales. These discrepancies are likely related to the weak rainfall variability exhibited by DePreSys3 and the fact that the main rainbelt does not extend far enough to the north (Fig. 10). We note that we do not compute re-cycling ratios or evaporation changes for the re-analysis data due to large uncertainties in re-analysis evaporation and precipitation estimates. Furthermore the moisture budget does not close for the re-analysis products [13].

Changes in the moist static energy terms for the two re-analysis products show similar features to DePreSys3 with increased specific humidity tending to de-stabilisize the lower atmosphere during wetter multi-year periods (Fig.

6 & Supplementary Figure 5). On inter-annual timescales only the upper atmosphere is de-stabilised by cooler temperatures, but unlike DePreSys3 this is also the case in the multi-year variability. As in DePreSys3, the inter-annual signal shows a strong cooling and less intense specific humidity increase at lower levels than the multi-year signal. Consequently, inter-annually, the lower atmosphere is not strongly de-stabilised during wet years.

Correlation maps between precipitation and global surface temperatures for re-analysis dataset are also consistent with the key regions identified as important for driving skilful Sahel rainfall change in DePreSys3: on multi-year timescales, a strong relationship exists between north Atlantic and Mediterranean SSTs and Sahelian summer rainfall in the re-analysis data, while the eastern tropical Pacific is significantly correlated with Sahel precipitation inter-annually (Supplementary Figure 6). However, as with the MSE and circulation patterns, differences between the two timescales are not as strong in the re-analysis data sets. Possible reasons are the use of an independent dataset to determine wet and dry composites (i.e. the GPCC observations), the inconsistencies between re-analysis products and weaker influences (e.g. from the Pacific) on multi-year timescales which DePreSys3 does not capture.

Supplementary Note 2: The African easterly jet, African easterly waves and Sahel rainfall in DePreSys3

The mid-altitude African Easter Jet (AEJ) is thought to influence Sahelian rainfall change [14–16]. In DePreSys3 and the re-analysis, we find that the AEJ does not appear to impact moisture convergence in the Sahel, which is dominated by the meridional flow (Figs. 4 & Supplementary Figure 4). However, it may play a role in dynamically triggering or enhancing rainfall events as discussed below.

Moisture advection into the Sahel by the mid-altitude African Easterly Jet (AEJ) shows a stronger modulation on multi-year compared to inter-annual timescales (Fig. 4). On multi-year timescales, increased Sahel rainfall corresponds to a strengthened AEJ as it shifts northward into the Sahel (Fig. 3c), tracking the meridional surface temperature gradient associated with the WAWJ and marine ITCZ [14]. It is interesting that AEJ moisture flux anomalies mostly counteract those from the lower level westerlies on these timescales (Fig. 4a). On inter-annual timescales, increased rainfall is coincident with a weakened AEJ anomaly which is centred to the south of the Sahel (Fig. 3e). This result supports other studies which show that increased Sahel rainfall reduces meridional surface temperature gradients required to establish an easterly flow at mid-altitudes [14,15].

Instabilities in the AEJ, known as African Easterly Waves (AEWs) can trigger, or be triggered by, Sahel rainfall events [17]. Here, we quantify AEWs by the mean summer cross-AEJ potential vorticity gradient, β, [16]. In DePreSys3, β is found to co-vary with precipitation across the Sahel on both inter-annual and multi-year timescales e.g. r = $O(0.9)$ in the western Sahel. However, it is difficult to disentangle causal relationships and feedbacks between AEWs and either Sahel rainfall, boundary layer temperature gradients or the TEJ which are all correlated in DePreSys3. In summary, although the position and strength of the AEJ does vary with Sahel rainfall levels, it does not strongly modulate moisture convergence in the region. However, it likely plays a role in intensifying rainfall events through AEW instabilities [16].

Supplementary References

1. Taylor, K.E., Stouffer, R. J. & Meehl, G. A. An overview of CMIP5 and the experimental design *Bull Am Meteoral Soc* **93,** 485–498 (2012).

2. Smith, D. M., Eade, R., Dunstone, N. J., Fereday, D., Murphy, J. M., Pohlmann, H & Scaife, A. A. Skilful multi-year predictions of Atlantic hurricane frequency *Nature Geoscience* **3,** 848–849 (2010).

3. Merryfield, W. J. et al. The Canadian seasonal to interannual prediction system. Part I: Modals and initialization. *Mon. Wea. rev. e-view* **141,** 2910–2945, doi:10.1175/MWR-D-12-00216 (2013).

4. Delworth, T. L. et al. GFDL's CM2 global coupled climate models. Part I: Formulation and simulation characteristics. *J. Clim.* **19,** 643–674, doi:10.1175/JCLI3629.1 (2006).

5. Jungclaus et al. Ocean circulation and tropical variability in the coupled model ECHAM5/MPI-OM. *J. Clim.* **19,** 3952-3972, doi:10.1175/JCLI3827.1 (2006).

6. Watanabe, M. et al. Improved climate simulation by MIROC5: Mean states, variability and climate sensitivity. *J. Clim.* **23,** 6312–6335, doi:10.1175/2010JCLI3679.1 (2010).

7. Kalnay et al. The NCEP/NCAR 40-year reanalysis project *Bull Am Meteoral Soc* **77,** 437–470 (1996).

8. Kobayashi, S., Ota, Y., Harada, Y., Ebita, A., Moriya, M., Onoda, H., Onogi, K., Kamahori, H., Kobayashi, C., Endo, H., Miyaoka, K. & Takahashi, K. The JRA-55 Reanalysis: General specifications and basic characteristics. *J. Meteor. Soc. Japan,* **93,** 5–48, :10.2151/jmsj.2015-001 (2015).

9. Harada, Y., Kamahori, H., Kobayashi, C., Endo, H., Kobayashi, S., Ota, Y., Onoda, H., Onogi, K., Miyaoka, K. & Takahashi The JRA-55 Reanalysis: Representation of atmospheric circulation and climate variability. *J. Meteor. Soc. Japan* **94,** 269–302, doi:10.2151/jmsj.2016-015 (2016).

10. Dee, D. P. The ERA-Interim reanalysis: Configuration and performance of the data assimilation system. *Quart. J. Roy. Meteor. Soc.* **137,** 553–507 (2011).

11. Trenberth, K. E. & Guillemot, C. J. Evaluation of the global atmospheric moisture budget as seen from analyses. *J. Climate.* **8,** 2255–2272 (1995).

12. Pomposi, C. P., Kushnir, Y., & Giannini, A. Moisture budget analysis of SST-driven decadal Sahel precipitation variability in the twentieth century. *Clim. Dyn.* **,** DOI: 10.1007/s00382-014-2382-3 (2014).

13. Seager, R. & Henderson, N. Diagnostic computation of moisture budgets in the ERA-Interim reanalysis with reference to analysis of CMIP-archived atmospheric model data. *J. Climate* **26,** 7876–7901 (2013).

14. Cook, K. Generation of the African easerly jet and its role in determining west African precipitation. *J. Climate.* **12,** 1165–1184 (1999).

15. Fontaine, B., Janicot, S. & Moron, V. Rainfall anomaly patterns and wind field signals over west Africa in August (1958–1989). *J. Climate* **8,** 1503–1510 (1995).

16. Vellinga, M., Roberts, M., Luigi Vidale, P., Mizielinski, M. S., Deomory, M., Schiemann, R., Strachan, J. & Bain, C. Sahel decadal rainfall variability and the role of model horizonal resolution *Geophys. res. Lett.* **,** DOI: 10.1002/2015GL066690 (2015).

17. Berry, G. J. & C. D. Thorncroff Case Study of an intense African easterly wave *Monthly Weather Review.* **133,** 752–766 (2005).
